# Coastal Upwelling Drives Intertidal Assemblage Structure and Trophic Ecology

**DOI:** 10.1371/journal.pone.0130789

**Published:** 2015-07-27

**Authors:** Carl J. Reddin, Felipe Docmac, Nessa E. O’Connor, John H. Bothwell, Chris Harrod

**Affiliations:** 1 School of Biological Sciences, Queen's University Belfast, Belfast, United Kingdom; 2 Instituto de Ciencias Naturales Alexander Von Humboldt, Universidad de Antofagasta, Antofagasta, Chile; 3 Institute of Global Food Security, Queen’s University Belfast, Belfast, United Kingdom; 4 School of Biological and Biomedical Sciences, Durham University, Durham, United Kingdom; University of Vigo, SPAIN

## Abstract

Similar environmental driving forces can produce similarity among geographically distant ecosystems. Coastal oceanic upwelling, for example, has been associated with elevated biomass and abundance patterns of certain functional groups, e.g., corticated macroalgae. In the upwelling system of Northern Chile, we examined measures of intertidal macrobenthic composition, structure and trophic ecology across eighteen shores varying in their proximity to two coastal upwelling centres, in a hierarchical sampling design (spatial scales of >1 and >10 km). The influence of coastal upwelling on intertidal communities was confirmed by the stable isotope values (δ^13^C and δ^15^N) of consumers, including a dominant suspension feeder, grazers, and their putative resources of POM, epilithic biofilm, and macroalgae. We highlight the utility of muscle δ^15^N from the suspension feeding mussel, *Perumytilus purpuratus*, as a proxy for upwelling, supported by satellite data and previous studies. Where possible, we used corrections for broader-scale trends, spatial autocorrelation, ontogenetic dietary shifts and spatial baseline isotopic variation prior to analysis. Our results showed macroalgal assemblage composition, and benthic consumer assemblage structure, varied significantly with the intertidal influence of coastal upwelling, especially contrasting bays and coastal headlands. Coastal topography also separated differences in consumer resource use. This suggested that coastal upwelling, itself driven by coastline topography, influences intertidal communities by advecting nearshore phytoplankton populations offshore and cooling coastal water temperatures. We recommend the isotopic values of benthic organisms, specifically long-lived suspension feeders, as *in situ* alternatives to offshore measurements of upwelling influence.

## Introduction

Environmental context has a large influence on ecosystem functioning [1–3], often setting the limits within which a general trend, such as the positive relationship between species richness and primary productivity [4], may hold. Understanding the influence of environmental context on communities is therefore a fundamental goal of ecology [5]. The relative importance of different environmental processes may vary among geographical regions [6] and with spatial scale [7], making their influence on communities difficult to predict at all but macroecological scales [8]. Fortunately, certain general scenarios, dominated by similar environmental conditions, appear to characterise multiple geographical regions (c.f. biomes [8, 9]). These merit investigation to identify the extent, and underlying driving processes, of ecological similarities (e.g. trophic structure [10]).

Coastal oceanic upwelling occurs where prevailing offshore winds create surface currents (eastern boundary currents), moving surface water offshore and drawing up cold, nutrient-rich waters from at depth [11]. This process characterises the California, Humboldt, Canary, and Benguela currents [11, 12], as well as many smaller systems (e.g. Galician [13]). These systems are associated with increased pelagic productivity because the nutrients that normally limit phytoplankton production become readily available, often creating phytoplankton blooms despite cooler water temperatures. Associated productivity and biomass can then support higher trophic levels (i.e. ‘bottom-up’ control [11,12]). Such oceanographic features are thought to have important effects on neritic and intertidal communities [14, 15] that can provide key ecosystem goods and services to human populations [16].

Upwelling effects on sublittoral and intertidal macroalgae have been observed at multiple scales. Among regions [17–20], and within regions over broad scales (100s–1000s km; Humboldt and Benguela Currents, Chile and South Africa [19, 20]) and intermediate scales (‘mesoscales’ hereon, 10s—100s km [12, 18, 20, 21]), with upwelling shores having increased macroalgal cover and biomass relative to non-upwelling shores. For example, in the South American Humboldt Current system, a higher biomass of low-intertidal kelps and corticated macroalgae (*Mazzaella* sp.) at shores near to centres of upwelling has been attributed to the increased availability of nutrients [18, 21]. In contrast, shores a short distance away from upwelling centres may have higher abundances of ephemeral macroalgae [18, 21–23]. This suggests that changes in macroalgal assemblages, including functional group predominance (e.g. corticated vs. ephemeral macroalgae), are predictable by proximity to upwelling centres and this may be a wider phenomenon.

Global similarities in the abundance structure of key functional groups (i.e. macrophytes, grazers, suspension feeders, predators) in intertidal communities suggest that macrophytes and suspension feeders dominate in terms of the relative amount of physical space occupied [10]. Yet at mesoscales these two functional groups may respond differently to proximity to upwelling centres. Menge et al. [12, 15] found that while macrophyte cover was highest at shores adjacent to upwelling centres in the California Current, higher abundances of suspension feeders, grazers and predators were recorded at more distant shores, potentially supported by higher water temperatures and phytoplankton concentrations. However, mesoscale upwelling conditions can vary temporally, as some centres of upwelling can strengthen, wane or shut down, the latter usually associated with ENSO status [24]. Such temporal dynamics in upwelling may be more important for community structure than proximity to upwelling centres [19]. Upwelling usually advects coastal water offshore, which can have important implications for the local population dynamics of broadcast spawning organisms (e.g. barnacle recruitment [25]) and planktonic populations [26]. Investigations of the complex relationships between intertidal communities and upwelling can be hindered by difficulties in defining a proxy for intertidal influence of upwelled waters [21]; in particular, an integrated measure over time-scales of ecological relevance [19].

Stable isotope analysis (SIA) provides a means to characterise ecosystem functioning, namely the origin of biologically available matter and energy flows through a community. This method uses two main features: firstly, the predictable differences in isotopic values of energy and nutrients originating from different sources (e.g. differences in δ^13^C values from primary producers of pelagic and benthic origin [27]; differences in δ^15^N associated on a shoreline with different parts of the nitrogen cycle [28, 29]); secondly, the predictable isotopic differences between consumers and their assimilated food (i.e. trophic fractionation [30]). In particular, the isotopic values of certain tissue types of long-lived consumers can serve as a temporally averaged estimation of the ‘isotopic baseline’ of the foodweb [31]. Spatial variation in δ^13^C and δ^15^N baseline, or ‘isoscapes’, for intertidal food webs have been demonstrated at various spatial scales (e.g. [32, 33]), however, broader scale studies of this nature are typically limited due to low replication (e.g. [21, 23]). Recently-upwelled C and N can be traced by ^13^C and ^15^N enrichment at the base of the food web, with primary producers utilising ‘new’ N tending to be isotopically heavier than those utilising recycled N from the excretions of consumers (e.g. [34, 35]).

During the current study, temporally and spatially integrated isotopic baselines provided by long-lived invertebrate consumers [31] were predicted to track the influence of freshly upwelled water in intertidal habitats. This was firstly confirmed by comparing spatial variation in observed isotopic baselines with contemporary satellite data [36], and the results of a previous key study in the region [23]. We then tested two hypotheses: (i) Isotopic indicators of upwelling correlate positively with (a) species richness and assemblage composition of primary producers and (b) diversity and assemblage structure of benthic consumers; and (ii) consumer resource use co-varies with the isotopic measure of upwelling intensity, with phytoplankton being more important at shores further from upwelling centres.

## Methods

### Study region

**Fig 1:**
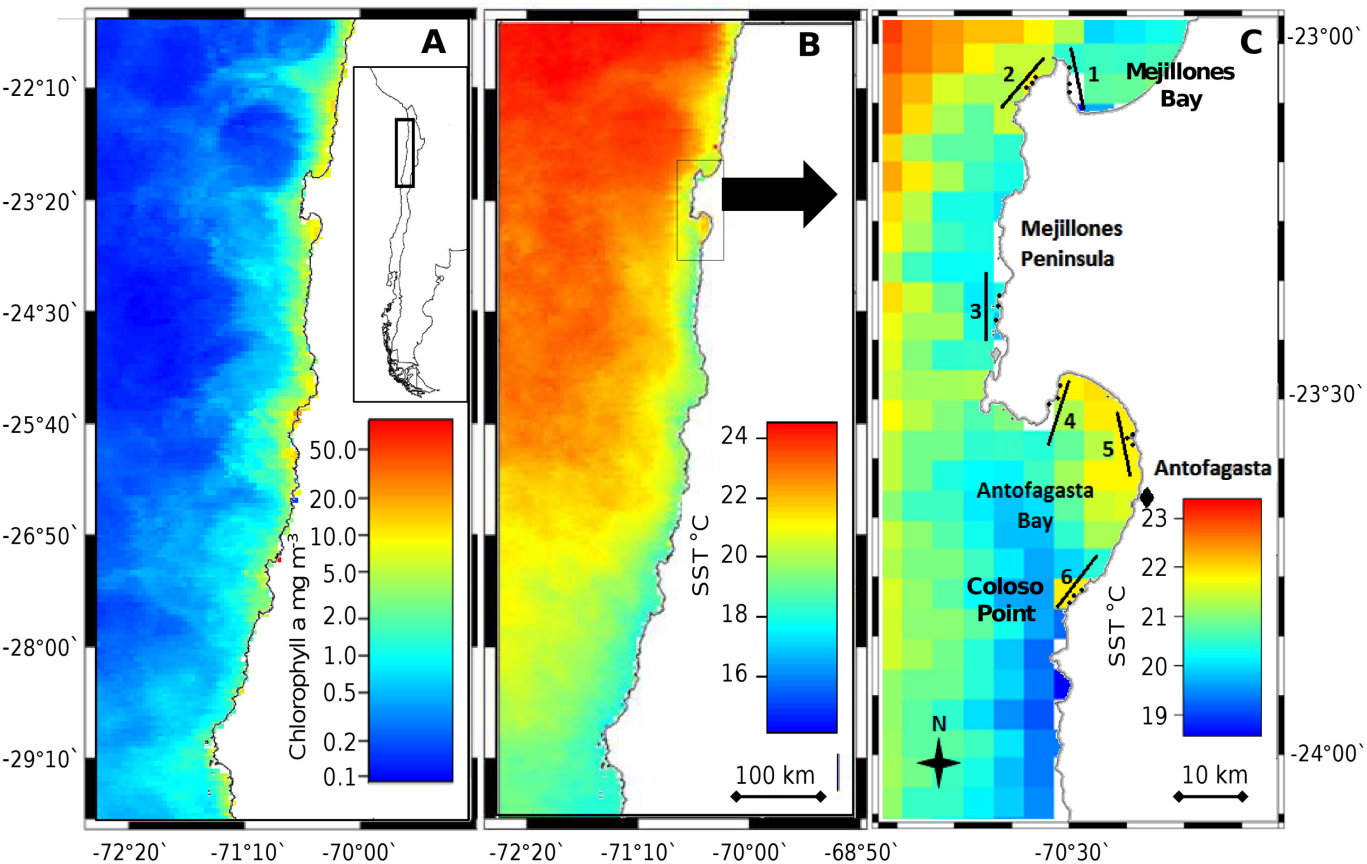
The anomalous warmth of Antofagasta Bay and upwelling context of the Mejillones Peninsula (arrow in map B) along the wider coastline of Northern Chile. (A) Coastal primary productivity (chlorophyll a concentration, logarithmic colour scale) and (B) cool upwelled water (sea surface temperature, SST) parallel to the coastline. (C) A ‘zoomed-in’ view of the Mejillones Peninsula with SST (note different temperature scale to ‘B’) shows ‘locations’ (10 km scale, black lines), ‘sites’ (1 km scale, small black points) and the city of Antofagasta (black diamond). All SST and chlorophyll a concentrations are mean values from estimated daily aqua MODIS satellite data [36] collected between December 2011 and February 2012 at a scale of 4.6 km. Euclidean distance from most northern to most southern sites was approximately 83 km. Where mentioned in the text, sites are numbered from ‘1’ to ‘3’ from north to south, nested within each location.

In the Humboldt Current region, locations of upwelling centres are generally determined by coastal topography [16, 37]. We focussed on the Mejillones Peninsula in the north of Chile, near the city of Antofagasta (approx. 23°20’ S, 70°30’ W; Fig 1). This region exhibits marked mesoscale variation in upwelling conditions [23, 38, 39], as shown by the austral summer 2011/2012 average sea surface temperature (SST; a standard proxy for upwelling intensity [18, 19, 23]) and surface chlorophyll a concentrations (Fig 1, see caption for data descriptions). The peninsula interrupts the otherwise almost linear coast of Northern Chile with its ca. 55 km-long face running parallel to the main coastline (Fig 1; also [40]). Antofagasta Bay is south-facing and receives prevailing north westerly winds, while on the peninsula’s leeward side, Mejillones Bay captures recirculated nutrient-rich waters from a local upwelling centre (an ‘upwelling shadow’ [37, 40]). Antofagasta Bay holds anomalously warm water for the region (Fig 1; also [40]) with SST > 17°C, with high primary productivity (Fig 1A), and water temperatures can surpass 20°C, e.g. at La Rinconada (location 4; see [41]), whilst outside the bay, mid-water temperatures average around 14°C, despite strong seasonal variability [41]. Mean tidal range around Antofagasta is 0.78 ± 0.14 m (mean over month of April 2015 [42]).

The intertidal communities of the Humboldt Current system are well described [22, 43, 44], with characteristic features including a low shore belt of encrusting lithothamnioid algae with stands of the large brown kelp *Lessonia nigrescens*, and dense beds of the mussel *Perumytilus purpuratus* on the mid shore [22]. In the warmer waters of Antofagasta Bay, however, both may be replaced by beds of the invasive ascidian *Pyura praeputialis*, though these have been subject to elevated levels of human exploitation [45]. Large grazers are often harvested by humans, but otherwise can be found from the mid shore downwards, including the chiton *Chiton granosus*, fissurelid and *Scurria* spp. limpets, and sea urchins [22]. The high shore is mainly dominated by the littorinid *Echinolittorina peruviana*, chthamaloid barnacles and ephemeral algae (mostly *Ulva* spp. [21]).

### Sampling structure

Six locations were selected (centred around 23°20’ S, 70°30’ W; See Fig 1C) to represent freshly upwelled and retained water conditions; five were those previously sampled in 2003 by Kelaher & Castilla [23], while the sixth was selected to provide a different bay setting, to aid the generalisation of analyses, and was situated to the north within Mejillones Bay. Proximity to upwelling was then validated using satellite derived SST and chlorophyll a data (Fig 1, data acquisition described in caption). Three sampling ‘sites’ (>1 km between central coordinates, ‘local-scale’), identified to summarise within-location rocky shore environmental region variation (compromised with site accessibility), were nested within six ‘locations’ (> 10 km between central coordinates, ‘mesoscale’). Field sampling was performed during February and March 2012, which was during the season of most intense upwelling.

### Field surveys

One 30-minute timed survey per site was used to rapidly estimate macroalgal species occurrence (presence/absence), consisting of an approximately 100 m wide vertical transect, from extreme high water to where sublittoral species dominated, covering all subhabitats present. Macroalgae were identified in the field or collected, along with larger fronds for examination for epiphytes, and returned to the laboratory for visual identification to the highest achievable taxonomic resolution using published keys [46, 47]. Surveys standardised by search time rather than area have been used extensively in cataloguing biodiversity [48]. Standardisation was improved by involving only one surveyor (to remove inter-observer differences [49]) and by practicing 10 times first, to minimise bias due to observer familiarisation with the technique and species [50]. Additionally, at each site, twelve 1 m^2^ quadrats were surveyed haphazardly for macrofauna species, using published keys [51, 52], ensuring that the high, mid, and low intertidal zones were all sampled. Macrofaunal abundances were then aggregated to represent the whole shore.

Environmental variation was described rapidly by ordinal values per site for substrate stability: (1) All bedrock; (2) mostly bedrock with sand or boulders; (3) some bedrock and some sand or mud with small boulders; (4) mostly rounded movable smooth boulders or sand; (5) all rounded movable smooth boulders or sand, and human activity (using the proxy of human settlement proximity, ‘near’ being < 1 km, ‘distant’ being > 1 km): (1) distant from paved road and settlements; (2) distant from paved road, small settlements nearby; (3) beach users nearby but large settlements distant; (4) paved road and settlements nearby; (5) urban coastline, per site. A third, mesoscale, ordinal variable was also used to account for effects associated with coastline topography other than upwelling (e.g. wave exposure, water temperature, long-shore current systems): (1) Inside a sheltered bay (locations 1, 4 and 5); (2) exposed coastline near the limits of a bay (location 6); (3) fully exposed headland (locations 2 and 3).

### Collection and preparation of specimens SIA

Dominant consumers were identified by pilot surveys. The mussel *P*. *purpuratus* and grazer *E*. *peruviana* are both conspicuous components of the northern Chilean intertidal community (mean density during the current study = 110 ± 318 and 57 ± 99 individuals m^-2^ respectively; note that the high level of variation was partly driven by the inclusion of quadrats representing high, mid and low shore). Additionally, because no large intertidal grazer was abundant at all sites, *Tegula atra*, a large, abundant sublittoral trochid gastropod, and *Scurria viridula*, an intertidal patelloid limpet that was frequently scarce, were selected for sampling (4 ± 22 and 1 ± 2 individuals m^-2^, respectively). Within each site, four individuals per species were collected where possible for subsequent analysis of δ^13^C and δ^15^N. Samples sizes at sites were small (n = 4) so the effect of ontogenetic dietary shift on isotopic values [53] was minimised by selecting individuals to target sizes, which were ca. 25 mm for *P*. *purpuratus*, 25 mm for *T*. *atra*, 12.5 mm for *E*. *peruviana*, and 40 mm for *S*. *viridula* (all measured on longest axis).

Samples of various putative food sources were also collected at each site: epilithic biofilm was scraped from rocks using a metal spatula, a 5 l water sample was collected for particulate organic matter (POM) and *Ulva* sp. fronds and dominant brown alga fronds (*Lessonia nigrescens*, or *Dictyota kunthii* where the former was absent) were collected. All samples were transported on ice, and frozen at -20°C at the laboratory, except water samples which were held at 4°C prior to prompt filtration.

Mollusc size was recorded as wet-mass (in shell; to 0.1 g) before muscle tissue (see [54]) was dissected out and washed with distilled water. Macroalgal tissue was taken from frond areas of recent growth. Water samples were well-mixed and filtered through pre-combusted (550°C for 4 hours) 0.7 *μ*m Whatman GF/F filters until they held enough mass for analysis, judged by a clear colour change. Being potentially carbonate-rich, epilithic samples were split in two (ca. 5 mg each), with one part decalcified by application of 10% hydrochloric acid drop-by-drop until bubbling ceased, to remove carbonate contaminates (for δ^13^C), and the other part remaining untreated (for δ^15^N [55]). The decalcified sample was then dried again and homogenised before being stored in a new container. All samples were oven-dried at 65°C for 48 h to a constant mass. Samples were standardised by dry mass to ca. 0.9 mg for fauna, 1.5 mg for *Ulva sp*. and 2 mg for *L*. *nigrescens* or *D*. *kunthii*, into tin capsules (6 * 4 mm, Sercon Ltd) on a Mettler Toledo XS3DU Microbalance, whilst GF/F filters were cut into sections. Samples were combusted in an elemental analyser coupled to a continuous-flow CHNOS Elemental Analyzer interfaced to an IsoPrime 100 mass spectrometer at the Center for Stable Isotope Biogeochemistry, University of California at Berkeley to estimate δ^13^C, δ^15^N, and elemental % C and % N values. Isotope ratio data were expressed in the standard δ unit, as the ratio of heavy to light isotopes, in ‰ units: δ (‰) = [Rsample / Rreference– 1] × 10^3^, with R = ^13^C/^12^C for carbon and ^15^N/^14^N for nitrogen. The standard for carbon is V-PDB. The standard for nitrogen is air. Two calibration standards were used; the external standard ‘peach leaves’, NIST SMR 1547, showed analytical precision to be 0.10‰ and 0.15‰ for δ^13^C and δ^15^N and an internal standard, *Patella vulgata* muscle, suggested precision to be < 0.1 ‰ for both δ^13^C and δ^15^N.

### Statistical analysis

Spatial variation in the isotopic patterns of resource and consumer groups was established by plotting standardised δ^13^C and δ^15^N (by subtracting the mean and dividing by the SD within each group). *Dictyota kunthii* and *L*. *nigrescens* were excluded owing to their geographical exclusivity to and from Antofagasta Bay (locations 4 & 5). Additionally, *S*. *viridula* was absent entirely from Mejillones Bay (location 1). We used spatial correlation to test for relationships between the isotopic patterns and SST values, the latter averaged over the geographically closest 4 km cells of SST data for each site.

Macroalgal composition (presence/ absence) was combined with isotopic ratios of *P*. *purpuratus* and a series of environmental variables through canonical correspondence analysis (CCA [56]; R package *vegan* [57]), with the outputs arranged in species-conditional triplots. CCA creates synthetic dimensions from a linear combination of given environmental variables, which maximises the niche separation amongst species. Species were treated to have a Gaussian rather than a linear response as in redundancy analysis; the analysis therefore approximated a multivariate Gaussian regression [58]. The triplot then displays sites and species against the two dimensions accounting for most variation. Permutation tests (9999 [58]) were used to test the significance of each CCA dimension.

Spatial correlations were tested using Spearman’s Rank or Pearson’s r. T-tests were used to compare abundances of consumer species between areas of high and low upwelling. Sites nearer to each other tended to be more closely related to each other than sites further away (spatial autocorrelation), indicating that spatial data were non-independent. Therefore, both non-spatial p-values and p-values derived from the Dutilleul et al. [59] method were reported. The latter test, available in the R package ‘Spatial Pack’ [60], accounts for the increased risk of Type I error., by calculating Moran’s I for spatial autocorrelation and adjusts the degrees of freedom appropriately.

Proportional dietary estimates of putative resources for consumers were estimated by Bayesian mixing models in the R package SIAR [61], performed separately for high and low upwelling areas as identified by site similarity in the isotopic baseline. Models used putative resources averaged within areas of high or low upwelling, while consumers were split into two groups; the suspension feeder (*P*. *purpuratus*) and grazers (*E*. *peruvina*, *S*. *viridula* and *T*. *atra*). SIAR is robust to unquantified sources of error [61]. Fractionation values for the intertidal mussel *Mytilus edulis* from Dubois et al. ([62] Δ^13^C = 2.2 ± 0.1, Δ^15^N = 3.8 ± 0.1) were used for all consumers.

The following assumptions of statistical analyses were examined: for spatial correlation tests, second order stationarity was tested using trend surface analysis (TSA [63]), although only *T*. *atra* C:N tested significant (Coefficient = -0.0011, p < 0.001); here the residuals were retained as the detrended variable. Putative ontogenetic dietary shifts in δ^13^C and δ^15^N were tested within species (pooled individuals), using ordinary least squares regression and individual wet-mass as a predictor. Despite size-targeting during collection, significant size co-variation was identified for δ^13^C in *E*. *peruviana* and *S*. *viridula* (positive slope), and δ^15^N in *E*. *peruviana* and *P*. *purpuratus* (negative slope; S1 Fig and S1 Table). Normality was assessed using histograms and the Shapiro-Wilk test. We decided *a priori* to transform non-normal data by the natural log or inverse to allow a Pearson’s correlation, otherwise Spearman’s Rank was used. Thus an inverse transformation was applied to *E*. *peruviana* size-corrected δ^13^C. All analyses were performed in the R statistical package [64].

## Results

**Fig 2:**
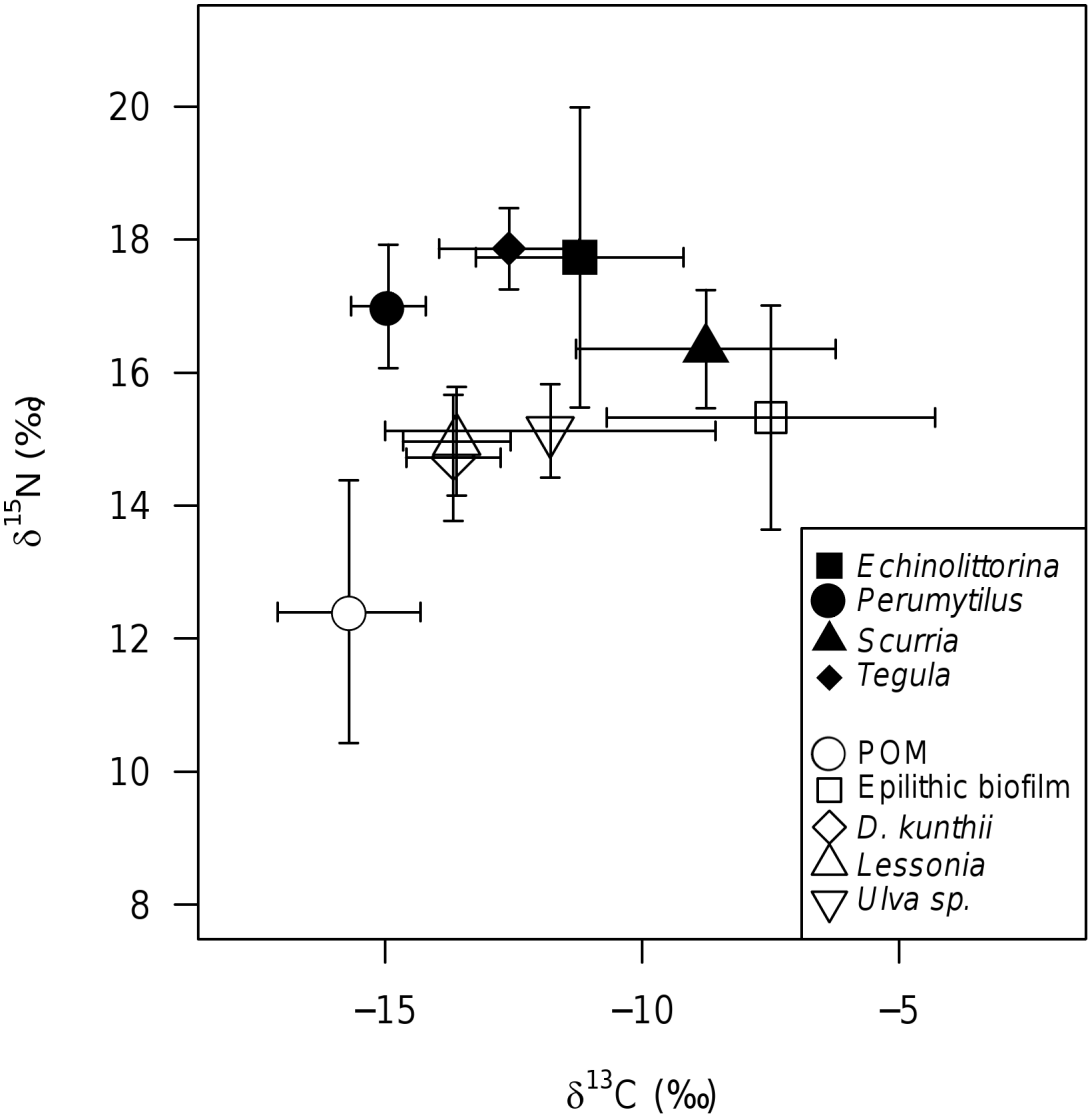
δ^13^C and δ^15^N isotopic composition (mean ± SD) of putative resources (open symbols) and consumers (closed symbols) along the coastline of Northern Chile. *D*. *kunthii* was present only at Antofagasta Bay (locations 4 & 5); *L nigrescens* was absent from Antofagasta Bay (locations 4, 5); *S*. *viridula* was absent from Mejillones Bay (location 1).

δ^13^C and δ^15^N values effectively discriminated the different primary producers (except perhaps *Ulva* sp.) and consumers (Fig 2), with primary producers varying in δ^13^C and consumers being ^15^N enriched relative to putative resources. Resources all had a similar δ^15^N (mean ± SD; 14.4 ± 1.9 ‰) except for POM, which was ^15^N depleted (12.4 ± 2.0 ‰) relative to the others. The suspension-feeding *P*. *purpuratus* was most ^13^C depleted (-14.9 ± 0.7 ‰) of the consumers, and had a δ^13^C very similar to POM (-15.7 ± 1.4 ‰; Fig 2). All consumer species mean C:N ratios were similar (3.3 ± 0.2), whilst putative resources had highly variable C: N ratios (epilithic biofilm = 8.1 ± 2.0, *D*. *kunthii* = 13.0 ± 1.3, *L*. *nigrescens* = 16.6 ± 1.7, *Ulva* sp. = 11.3 ± 2.0).

### Spatial variation in isotopic baseline: an intertidal signature of upwelling

**Fig 3:**
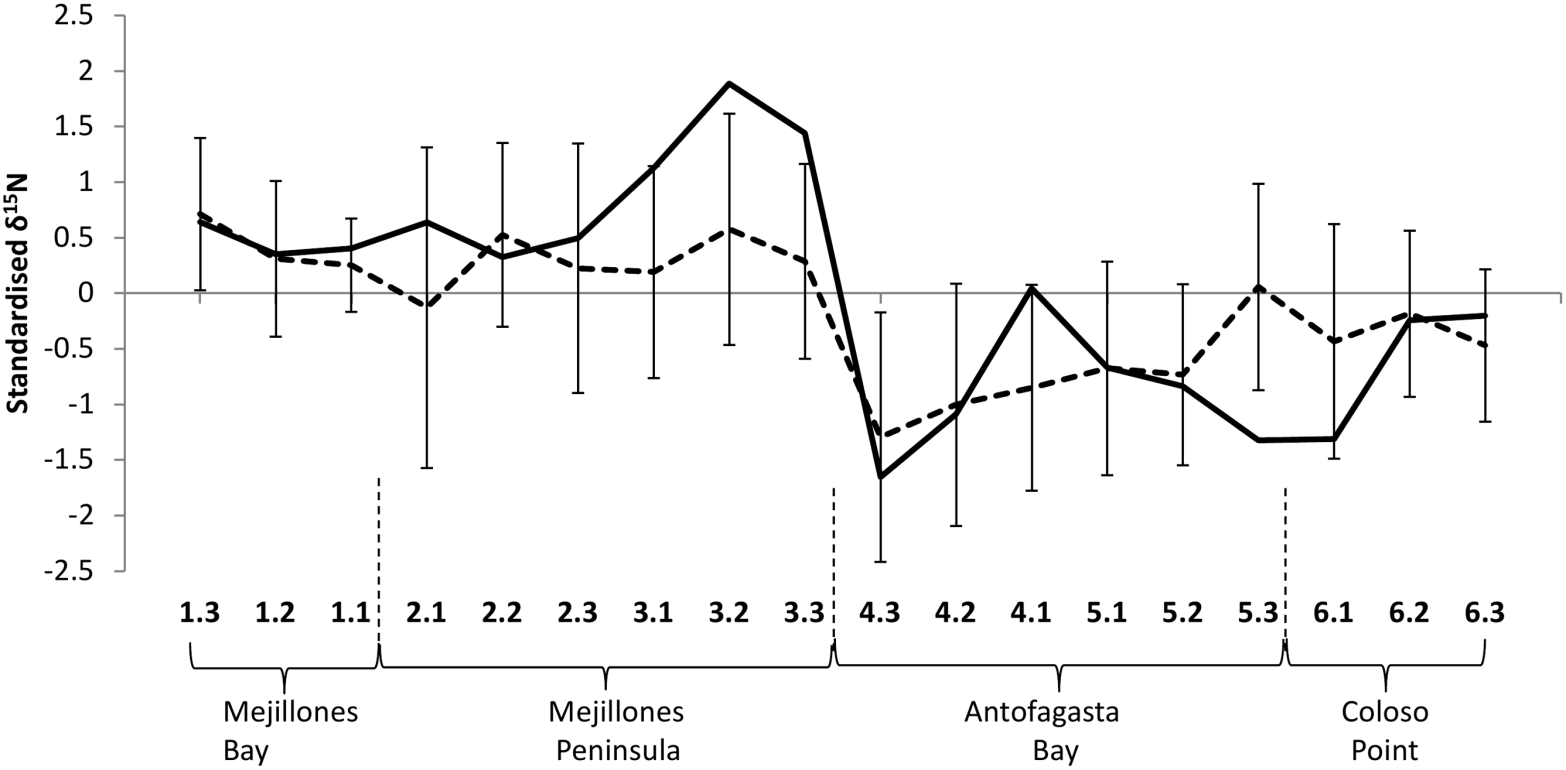
Geographical variation of standardised δ^15^N at sites along the coastline. The solid line shows size-corrected *P*. *purpuratus* δ^15^N, whilst the dashed line shows the mean (± SD) across standardised δ^15^N of all consumers (*P*. *purpuratus*, *E*. *peruviana*, *S*. *viridula*, *T*. *atra*) and putative resources (POM, epilithic biofilm, *Ulva* sp.). Site labels are presented below the graph in sequence around the coast, with the main geographical features summarised at the base of the graph (see Fig 1 for more detail).

All consumers and putative resources (‘mean’ line with error bars in Fig 3) were relatively ^15^N enriched in Mejillones Peninsula and Mejillones Bay (locations 1–3). Antofagasta Bay and Coloso Point (locations 4–6) were relatively ^15^N depleted (Fig 3). Size-corrected δ^15^N values of the suspension feeding mussel, *P*. *purpuratus*, represented the ^15^N enrichment trend well, fitting assumptions of its validity as a pelagic resource baseline, and was used to represent general δ^15^N baseline from here on. Apparent agreement of the δ^15^N baseline with satellite-derived SST patterns (Fig 1 and caption) was validated statistically (Spearman’s Rho = -0.55, DF = 23.3, spatial p = 0.01), indicating that intertidal δ^15^N values reflect upwelling intensity. Coastal upwelling did not appear to underlie spatial variability in δ^13^C (mean across consumers and putative resources, and *P*. *purpuratus*; Spearman’s Rho = 0.21, DF = 10.5, spatial p = 0.69), which was highly variable within locations.

### Upwelling influence and community composition: macroalgae and consumers

**Fig 4:**
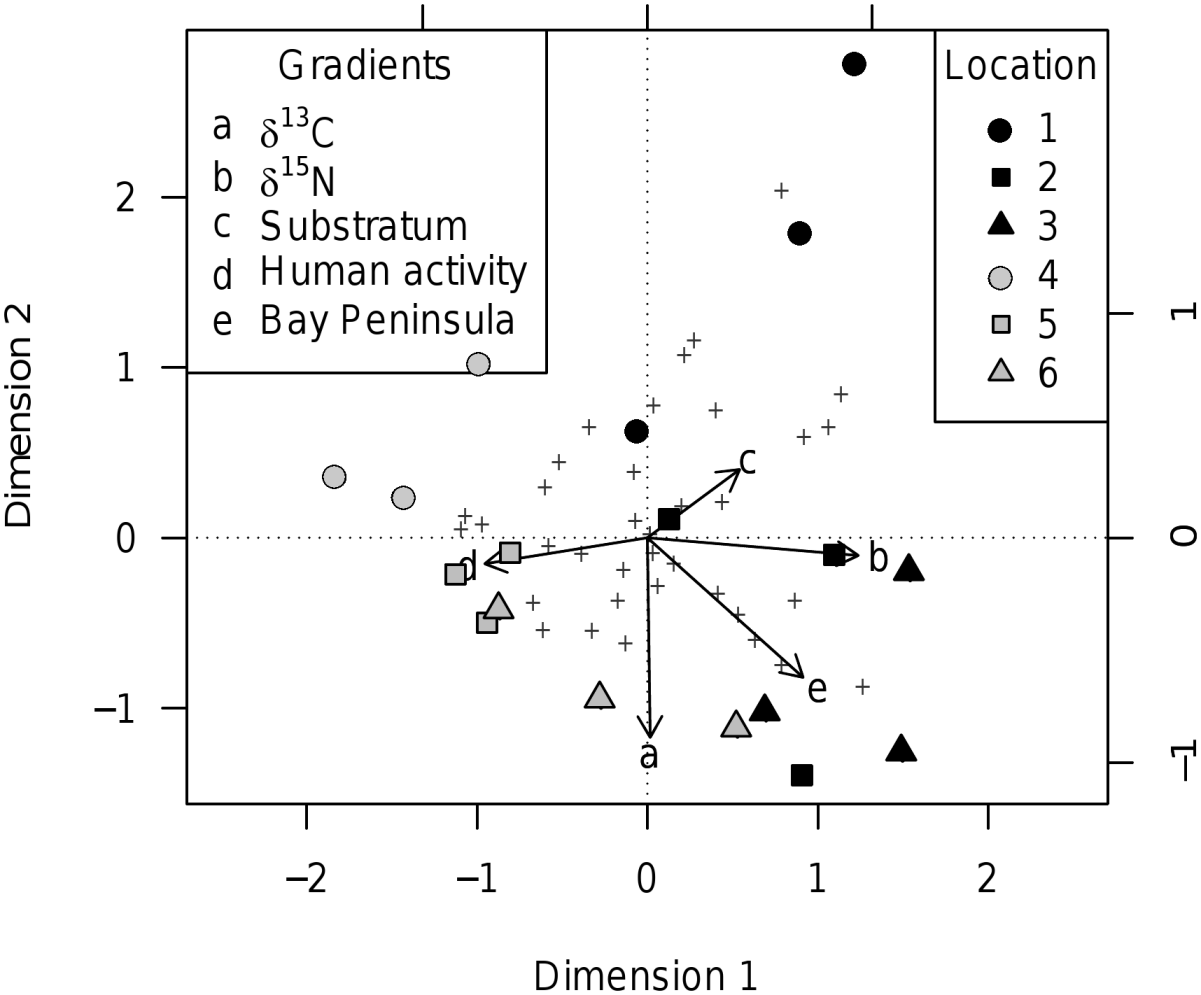
The distribution of sites (symbols) and macroalgal species (crosses) over environmental gradients (arrows), showing the separation of Antofagasta Bay from Mejillones Peninsula. A species-conditional triplot based on a canonical correspondence analysis, with *P*. *purpuratus* δ^13^C and δ^15^N included as environmental gradients. Eigenvalues of dimension 1 (horizontal) = 0.20 and dimension 2 (vertical) = 0.19; eigenvalue of the axis 3 (not displayed) = 0.09. Scale marks along the axes apply to the species and sites scores. Species crosses represent the weighted average of their ‘niche’ (by site), though labels were omitted to avoid cluttering the plot (listed in S2 Table). Rare species which occurred at <2 sites were removed *a priori* to analysis, as recommended by Bocard et al. [58]. Site symbols show Mejillones Peninsula (locations 2 & 3) and Bay (location 1) in black, and Antofagasta Bay (locations 4 & 5) and Coloso Point (location 6) in grey. 2D triplot displays 26.9% of total inertia (= weighted variance) in the observed occurrences and 65.3% of variance in the weighted averages and class totals of macroalgal species with respect to the environmental variables.

Macroalgal occurrence patterns significantly contrasted sites of high upwelling influence (*P*. *purpuratus* δ^15^N as proxy, biplot score = 0.94; Fig 4) and sites of higher human activity (biplot score = -0.72; both variables contrasted by CCA dimension 1, Eigenvalue = 0.2, F-value = 2.8, p < 0.01, 9999 permutations, full details in S2 Table) and, secondarily, associated significantly with *P*. *purpuratus* δ^13^C (biplot score = -0.89; Fig 4; CCA dimension 2, Eigenvalue = 0.19, F-value = 2.68, p < 0.001, 9999 permutations; other dimensions were insignificant, S2 Table). All the Antofagasta Bay sites clustered together, as did the Mejillones Peninsula sites. Macroalgal species scores against CCA dimensions 1 and 2 are listed in S3 Table, including *L*. *nigrescens* (CCA1 = 0.53, CCA2 = -0.46) and *D*. *kunthii* (CCA1 = -0.61, CCA2 = 0.29). The CCA triplot represented a reasonably faithful representation of the multidimensional structure of the macroalgal occurrence data (by variance in the weighted averages and class totals, and by total inertia displayed, see Fig 4 caption).

Pairwise correlations between consumer isotopic gradients and macroalgal species richness, consumer Simpson’s D, and consumer abundances revealed no consistent correlations across consumer species (Table 1). Macroalgal richness was significantly associated with isotopic values of the subtidal trochid *T*. *atra*, with brown macroalgal richness being positively correlated (r = 0.59, spatial p < 0.05) with *T*. *atra* δ^13^C, and green macroalgal richness being correlated (r = 0.56, non-spatial p < 0.05) with *T*. *atra* δ^15^N. *P*. *purpuratus* δ^15^N values were negatively correlated with *P*. *purpuratus* abundance (r = -0.74, non-spatial p < 0.001), while the limpet *S*. *viridula* δ^15^N was positively correlated with *S*. *viridula* abundance (r = 0.65, non-spatial p < 0.05).

**Table 1:** Local-scale correlations between consumer isotopic values (δ^13^C and δ^15^N), macroalgal richness components, and consumer diversity and abundance of the species.

	δ^15^N	Macroalgal richness	Green macroalgal richness	Brown macroalgal richness	Red macroalgal richness	Consumer Simpson’s *D*	Local abundance
**A)** *P*. *purpuratus*							
δ^13^C	0.20	0.39	-0.30	0.25	0.19	-0.30	-0.20
δ^15^N^C^	1	0.19	-0.12	-0.34	0.42	0.27	-0.74(***)
**B)** *E*. *peruviana*							
δ^13^C^C^	-0.02	-0.05	0.28	-0.04	-0.10	-0.22	0.13
δ^15^N^C^ (inverse)	1	0.41	0.0	0.30	0.09	-0.04	-0.19
**C)** *S*. *viridula*							
δ^13^C^C^	0.36	0.19	-0.06	-0.44	0.27	0.04	0.30
δ^15^N	1	-0.16	-0.23	0.06	-0.01	0.02	0.65(*)
**D)** *T*. *atra*							
δ^13^C	-0.31	0.04	-0.35	0.59(*)	-0.26	-0.18	0.20
δ^15^N	1	0.20	0.56(*)	0.03	0.11	-0.17	-0.14

Consumers A) *P*. *purpuratus*, B) *E*. *peruviana*, C) *S*. *viridula* and D) *T*. *atra*, and correlations are Pearson’s r, or Spearman’s r for green and brown richness and all local abundances. Superscript ‘c’ on the δ^13^C and δ^15^N denotes that the values have been corrected by size in attempt to remove putative ontogenetic shifts in trophic habits. Sites n = 18 for *P*. *purpuratus* and *E*. *peruviana*; n = 12 for *S*. *viridula*; n = 14 for *T*. *atra*. P-values shown as

*** P < 0.001,

*P < 0.05;

d.f. corrected by Dutilleul et al. [58] method; values in brackets are standard non-spatial tests.

Correlations of the abundances of dominant taxa (i.e. those with potentially high local substratum coverage or abundance: barnacles, *P*. *praeputialis*, *P*. *purpuratus*, *E*. *peruviana*, *T*. *atra*, *S*. *viridula*; ecosystem engineers: *L*. *nigrescens*, *P*. *praeputialis*; and the predator *H*. *helianthus*) revealed two distinct assemblages (Table 2). *E*. *peruviana*, *P*. *preaputialis*, *P*. *purpuratus* and barnacles were significantly positively associated, representing Antofagasta Bay, and negatively associated with *H*. *helianthus*, *S*. *viridula* and the kelp *L*. *nigrescens*, representing the outer Mejillones Peninsula. Moreover, the abundances of organisms of these distinct assemblages were significantly associated with the intertidal proxy of upwelling influence (*P*. *purpuratus* δ^15^N; Table 2). When the abundances of dominant taxa were averaged exclusively over Mejillones Peninsula (locations 2–3) and Antofagasta Bay (locations 4–5), some taxa were significantly different (*E*. *peruviana*, non-spatial p < 0.01; *P*. *purpuratus* and *H*. *helianthus*, both non-spatial p < 0.05; Table 3).

**Table 2:** The spatial separation of two distinct assemblages of dominant taxa.

Putative trophic functional group	Grazer	Grazer	Suspension feeder	Suspension feeder	Suspension feeder	Predator	Grazer	Kelp
Species (abundance of)	*T*. *atra*	*E*. *peruviana*	*P*. *purpuratus*	*P*. *preaputialis*	Barnacle %	*H*. *helianthus*	*S*. *viridula*	*L*. *nigrescens*
**Upwelling influence^a^**	-0.11	-0.74(***)	-0.74(***)	-0.73(***)	-0.42	0.19	0.68(**)	0.50(*)
*T*. *atra*	—	0.24	0.31	-0.08	-0.07	-0.15	0.34	-0.26
*E*. *peruviana*		—	**0.75**(***)	**0.69**(**)	**0.51**(*)	-0.19	-0.51(*)	-0.30
*P*. *purpuratus*			—	**0.59**(**)	**0.50**(*)	-0.41	-0.48(*)	-0.54(*)
*P*. *preaputialis*				—	**0.51**(*)	-0.31	-0.71(***)	-0.59(**)
Barnacle %					—	-0.20	-0.45	-0.17
*H*. *helianthus*						—	**0.15**	**0.69**(***)
*S*. *viridula*							—	**0.24**

Spatial co-occurrence (positive correlations, bold) and separation (negative correlations) of taxa by abundance (n.b. presence/ absence for *L*. *nigrescens*), and taxa abundance associations with upwelling influence (^a^ *P*. *purpuratus* δ^15^N). All correlations are Spearman’s Rank with non-spatial p-values shown in brackets as

*** P < 0.001,

** P < 0.01,

*P < 0.05.

For clarity, only one half of the symmetrical correlation matrix has been included.

**Table 3:** Spatial differences in abundance of dominant taxa between the outer Mejillones Peninsula (^b^ locations 2 and 3) and Antofagasta Bay (^c^ locations 4 and 5).

	Mean abundance (ind. m^-2^) ± SD		
	Outer peninsula^b^	Inner bay^c^	t-test P value
*T*. *atra*	0.04 ± 0.05	9.90 ± 17.02	ns
*E*. *peruviana*	10.94 ± 3.18	**89.42 ± 44.82**	(**)
*P*. *purpuratus*	0.17 ± 0.3	**199.83 ± 186.25**	(*)
*P*. *preaputialis*	0 ± 0	**26.58 ± 55.13**	ns
Barnacle %	9.22 ± 8.33	**26.86 ± 22.28**	ns
*H*. *helianthus*	**0.88 ± 0.58**	0.13 ± 0.13	(*)
*S*. *viridula*	**0.96 ± 1.34**	0.05 ± 0.1	ns
*L*. *nigescens*	**6/6**	0/6	-

P-values derived from a non-spatial two-tailed t-test,

** P < 0.01,

*P < 0.05,

ns = not significant. Proportion of sites present at, for *L*. *nigrescens*.

### Spatial variation in consumer resource use

**Fig 5:**
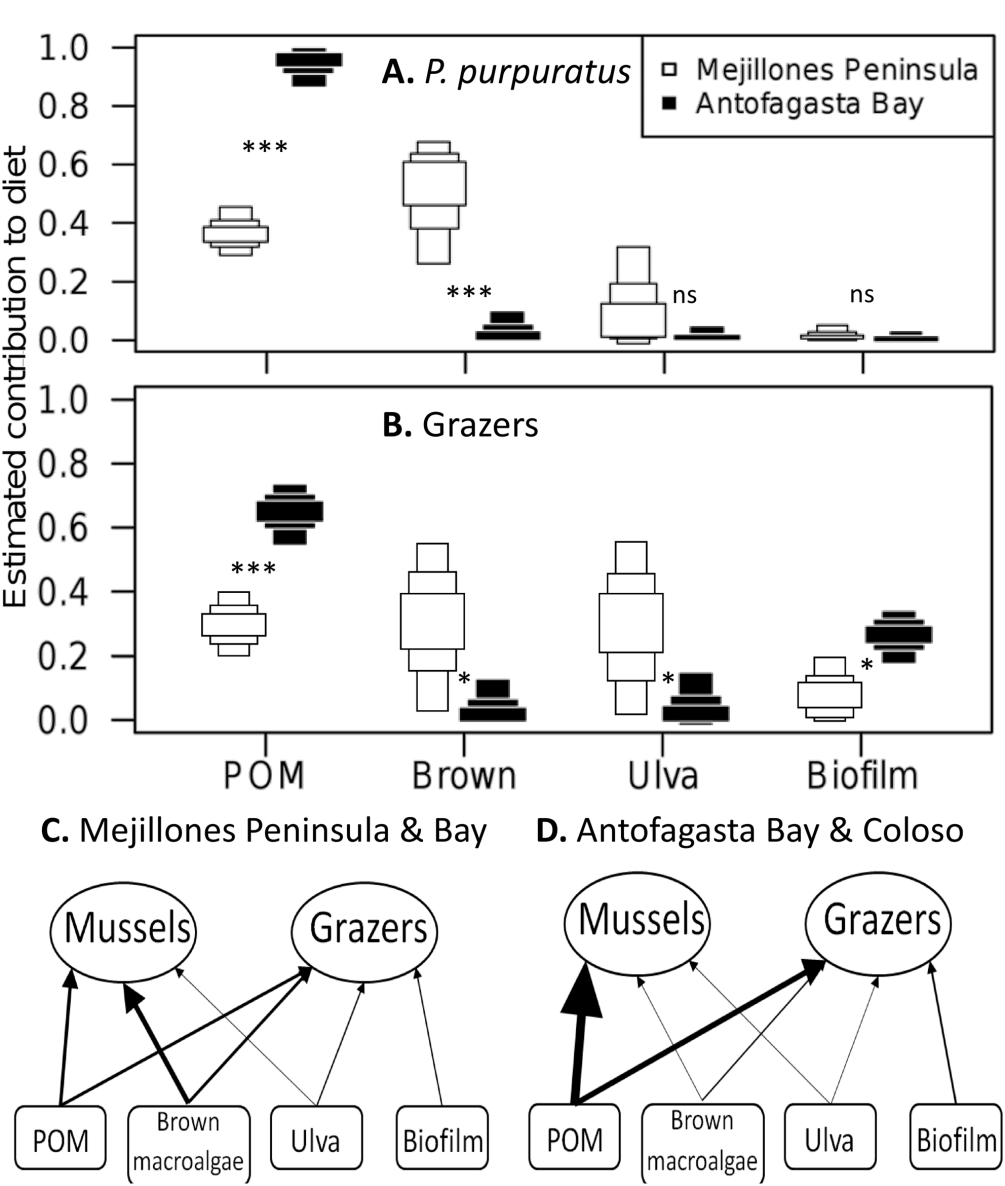
The geographical switching in importance of POM and brown macroalgae to the diets of intertidal consumers. Dietary contributions by resources to (**A**) the mussel *P*. *purpuratus* and (**B**) grazer species together, estimated by SIAR mixing models run separately for Mejillones Peninsula and Bay (locations 1–3), and Antofagasta Bay with Coloso Point (locations 4–6). Plotted are the 95, 75 and 50% Bayesian credibility intervals, with significance of differences between peninsula and bay estimates. ‘Brown macroalgae’ represents *L*. *nigrescens* and *D*. *kunthii*, which were combined due to isotopic similarity (Fig 2). The food webs of (**C**) Antofagasta Bay and (**D**) Mejillones Peninsula plotted figuratively. Arrow weight represents dietary importance by SIAR proportion estimates (mode).

Mixing models revealed considerable spatial differences in invertebrate consumer putative diet between Mejillones Peninsula and Bay (locations 1–3), and Antofagasta Bay with Coloso Point (locations 4–6) in both the suspension feeding *P*. *purpuratus* and, to a lesser extent, pooled grazer species (Fig 5). Consumer diet was significantly dominated by POM in Antofagasta Bay (suspension feeder mode = 0.96, 95% CI = 0.88–0.99; grazer mode = 0.66, 95% CI = 0.55–0.73) relative to the Mejillones Peninsula (suspension feeders = 0.36, 95% CI = 0.28–0.45, probability of difference > 99.9%; grazers = 0.3, 95% CI = 0.2–0.4, probability > 99.9%). Macroalgae was significantly more important to the putative diet of both suspension feeders (brown macroalgae mode = 0.56, 95% CI = 0.26–0.68, probability > 99.9%) and grazers (mode = 0.34, 95% CI = 0.02–0.55, probability > 95%), on Mejillones Peninsula relative to Antofagasta Bay. Close isotopic overlap between brown macroalgae and *Ulva* caused problems for the Mejillones Peninsula mixing model, especially for *P*. *purpuratus*. However, re-running the model with the two macroalgae pooled *a priori* gave a similar proportional estimate (mode = 0.60, 95% CI = 0.49–0.68) to previous estimates for brown macroalgae, for *P*. *purpuratus*.

## Discussion

Organism δ^15^N can provide information on the assimilation of N originating from a range of sources, including upwelled, ‘new’ N (^15^N enriched) from the assimilation of inshore waters’, which contrasts with ^15^N depleted ‘recycled’ N. As such, δ^15^N patterns from consumers and their putative resources were hypothesised to trace spatial variation in upwelling influence. Patterns in our δ^15^N data statistically matched contemporary satellite SST data and showed a general agreement with mesoscale variation in upwelling observed at the turn of the previous century [23].

### Upwelling influence and intertidal consumer resource use

We identified evidence that at the time of sampling (Summer 2012), the relative contribution of POM to consumer diets was reduced, and assimilation from macroalgae increased over the Mejillones Peninsula and Bay relative to Antofagasta Bay and Coloso Point. This geographical split was supported by a shift in the values of *P*. *purpuratus* δ^15^N, our *in situ* proxy of upwelling influence on intertidal habitats. This difference in resource use was particularly clear for mussels (see also [65]), despite a consensus that mussels selectively consume phytoplankton [13, 66] over particulate materials originating from other primary producers, such as kelp. Phytoplankton supply might therefore have been limited inshore at Mejillones Peninsula, forcing intertidal consumers to utilise alternate food sources [67]. The proposal of relative phytoplankton depletion at Mejillones Peninsula was supported by long-term chlorophyll a patterns, although satellite derived data may perform less well with increased proximity to the coast [68]. Meanwhile, in Antofagasta Bay the mussel *P*. *purpuratus* appeared to consume a more pelagic-derived diet (POM, presumably including phytoplankton) despite the occasional presence of kelp and other brown macroalgae, supporting its preference for phytoplankton. Finally, the coincidence of a more benthic diet (putative consumption of macroalgae) and low physiological condition was suggested by the occurrence of low *P*. *purpuratus* C:N values, a proxy for muscle-lipid content (e.g. [69]), at highly upwelling-influenced sites (*P*. *purpuratus* C:N correlated with δ^15^N, r = -0.46, non-spatial p < 0.05). Low mussel growth coinciding with low phytoplankton concentrations has been recorded on a shore adjacent to an upwelling centre by Menge et al. [15]. This paradoxical concurrence of high nutrient levels with low phytoplankton levels was attributed to the combination of low temperature of upwelled water [70] and rapid offshore transport, meaning that, by the time phytoplankton abundance had increased, populations had been advected offshore, away from the mussels [14]. Cool water temperatures, offshore advection and high nutrients may therefore increase the importance of macroalgal matter for intertidal consumers at shores close to upwelling centres [71–74]. Conversely, in Antofagasta Bay, where coastal topography leads to the retention of upwelled waters and allows them to warm, ideal conditions for phytoplankton populations were established; these fertile waters can then support large suspension feeder populations and biomass.

Where our mixing models identified a benthic dietary contribution to our consumers, we refrain from suggesting any specific macroalgal species was being preferentially consumed, especially by suspension feeders; indeed it is more likely that macroalgal species contributions to the particulate matter resource pool might be relative to their local biomass [71].

### Changes in intertidal consumer resource use and dominant functional group

Benthic community structure can be considered to represent a time-integrated signal of nearshore oceanographic conditions (e.g. [19]). Among the sites in this study, we observed differences in community structure to be driven by two distinct assemblages. These were characterised by negative associations between the abundances of *E*. *peruviana*, *P*. *praeputialis*, *P*. *purpuratus* and barnacles on the one hand, typifying Antofagasta Bay, and of *Heliaster*, *S*. *viridula* and *L*. *nigrescens* on the other, typifying the outer Mejillones Peninsula. Abundance patterns of these species correlated significantly with *P*. *purpuratus* δ^15^N, our proxy for intertidal upwelling influence. Species functional groups confirmed a suspension-feeder dominated community in Antofagasta Bay (e.g. correlation between *P*. *purpuratus* δ^15^N values and barnacle, the ascidian *P*. *praeputialis*, and *P*. *purpuratus* abundances). These results were analogous to the findings of Menge et al. [12, 14–15] in Oregon, with the outer Mejillones Peninsula resembling Boiler Bay, and Antofagasta Bay resembling Strawberry Hill (these Oregon sites were shown to be regionally representative in Menge et al. [15]). Covering both the Beneguela and Humboldt Current regions, Wieters et al. [19] found associations between within-functional group abundance, including suspension feeders, and temporal components of SST variation. However, after documenting ‘strikingly different’ temporal dynamics of near shore oceanographic conditions between the regions, they also stress that the similarity of different regions is not to be assumed [19]. Human interference, such as gleaning of large gastropods (e.g. limpets), can vary according to regional traditions and can be strong enough to impact community structure (e.g. the Chilean intertidal [75, 76]). However, the relative abundance of different functional groups can be remarkably similar among upwelling regions [10, 17, 19]. Our study suggests functional group abundance patterns may vary at a spatial scale corresponding to coastline topography, with coastal features that retain surface waters (e.g. bays), potentially promoting higher phytoplankton concentrations, higher consumer abundances (e.g. suspension feeders) and intertidal recruitment [26].

Macroalgal assemblage composition was also associated with variation in *in situ* upwelling influence, and clearly contrasted between the outer Mejillones Peninsula and Antofagasta Bay. Macroalgal assemblage differences are more likely to be related to abiotic conditions, particularly nutrients and temperature rather than variation in phytoplankton concentrations, as suggested above for intertidal macro-invertebrate consumers. Mejillones Bay, for example, was dissimilar to all other locations. Although elevated temperatures relative to surrounding waters have been recorded here [26], seasonally averaged SST indicated that long-term water temperature was likely to be cooler than that of Antofagasta Bay. When Castilla [77] experimentally transplanted the kelp *L*. *nigrescens* to Antofagasta Bay, thalli showed reductions in growth, survival and photosynthesis relative to control-transplanted thalli. Although not explicitly linked to temperature, Castilla [77] suggested that the kelp’s absence from Antofagasta Bay might reflect an unfavourable environment in the bay. In agreement with SST maps, the presence of *L*. *nigrescens* at Mejillones Bay site 1, and throughout Coloso Point sites, might indicate cooler water temperatures than in Antofagasta Bay.

### A stable isotope approach for recording upwelling influence

A key advantage of the stable isotope approach for understanding the origin and flow of energy and nutrients through a system is that patterns are integrated over extended temporal scales, owing to the averaging effect of consumer tissue turnover. Short-term changes in upwelling can be detected by including taxa, or tissues, that assimilate energy and nutrients over different time periods prior to sampling [35]. At the time of sampling in Sumer 2012 POM, for example, was isotopically lighter relative to benthic resources in both C and N [13, 27], but was strikingly depleted in ^15^N relative to the putative specialist filter feeder. A shorter turnover for POM relative to longer-lived resources (e.g. macroalgae) might then suggest this depletion to demonstrate a short-term decrease in enriched, upwelling-derived N. The difference between fractionation-corrected δ^15^N values from a putative consumer of POM (*P*. *purpuratus*; using the mytiloid fractionation values of Dubois et al. [62]) and sampled POM also supports a short-term decrease in upwelling. The long-term validity of our samples of POM is therefore uncertain, which could have affected the contribution estimates for this resource. Stable isotope analysis makes use of variation in tissue turnover and has been used previously to assess temporal variation in upwelling intensity [35, 78]. This serves as a reminder of the importance of temporal variation in upwelling [18], at multiple scales, and its effect on near-shore communities [19, 70].

In this study, muscle δ^15^N of the regionally ubiquitous mussel *P*. *purpuratus* was used as a proxy for intertidal influence of upwelling, and closely represented spatial patterns in wider organism δ^15^N. Bivalve muscle turnover rates have been reported at every three to six months [54]. *P*. *purpuratus* muscle should therefore reflect an average of its assimilated resource δ^15^N (plus trophic fractionation) over a similar period. Moreover, mussels make an excellent *in situ* baseline for pelagic processes [31, 79], in our case the intertidal influence of coastal upwelling. A recent approach, allowing further clarification of the geographical signal in isotopic baseline estimates, has used compound-specific isotopes (e.g. amino acids [80]). Spatial patterns in organism δ^13^C were inconsistent in this study and not statistically correlated to upwelling [34]. Environmental temperature may affect diet-consumer trophic fractionation in ectothermic organisms [81]. Extrapolating from Barnes et al. [81], the temperature difference between bay and outer peninsula situations (~3°C) predicts an enrichment of 0.3 ‰ for both ^13^C and ^15^N, which, being close to the level of analytical error (± 0.1 ‰), in this case is negligible.

The coastal upwelling plumes off the Mejillones Peninsula are amongst the most temporally persistent in the SE Pacific [38], sustaining high primary productivity throughout the year [39]. Still, organism δ^15^N values from Coloso Point suggested upwelling intertidal influence to be relatively low, agreeing with our 4 km grain SST maps but differing from previous SST observations [23] from 1997–8. This period was an El Niño phase whereas our study fell during a La Niña phase [82]. Inter-annual differences might, therefore, underlie the differences in encountered upwelling conditions. Water temperatures can be up to 5°C warmer during El Niño than other years [41], reducing the intensity of upwelling at known centres or even the cessation of upwelling at such locations [24]. The standard proxy for upwelling intensity is long-term SST, having a strong negative correlation with both nitrate concentrations and chlorophyll a maximum [18, 19, 23, 83]. However, this difference highlights the importance of appropriately dated SST data for predicting upwelling [18].

### Conclusions

This study confirmed the isotopic values of intertidal organisms, especially suspension feeding mussels [72, 80], as an *in situ* measure of upwelling influence. We identified spatial patterns at a high spatial resolution in macroalgal assemblage composition and consumer assemblage structure and found them to be significantly associated with mesoscale patterns in upwelling influence in the Humboldt Current system [18, 19, 21]. These results echoed relationships found in the Benguela [19, 20, 72, 73] and California Currents [12, 15]. A potential mechanism underlying these community differences was suggested by consumer resource use, that limited phytoplankton availability coinciding with high nutrients might favour large macroalgae (e.g. kelp) at shorelines adjacent to upwelling centres. In this context, macroalgae may become important resources for local consumers. Conversely, shorelines that retain waters upwelled from nearby tend to be dominated by suspension feeder assemblages, where consumers may take advantage of the warm, productive waters. It is possible that these two scenarios may represent points along a general trend for intertidal zones on upwelling coastlines. Variation in the proximity of intertidal communities to active upwelling centres can be produced by coastal topography (e.g. [12]), as exemplified in Northern Chile by the Mejillones Peninsula and Antofagasta Bay [37]. We therefore recommend further study of upwelling influence on intertidal community dynamics to include spatial scales reflecting coastal topography, and temporal scales reflecting oceanographic cycles [18, 19].

## Supporting Information

S1 Fig Ontogenetic isotopic shifts in consumers. Significant coefficients between individual mass and *E*. *peruviana* δ^13^C and δ^15^N (A & B, respectively), and *S*. *viridula* δ^13^C (C), and *P*. *purpuratus* δ^15^N (D). Regression residuals were normal.
(TIF)

S1 Table Ontogenetic isotopic shifts in consumers. Significant coefficients between individual mass and *E*. *peruviana* δ^13^C and δ^15^N (A & B, respectively), and *S*. *viridula* δ^13^C (C), and *P*. *purpuratus* δ^15^N (D).
(DOCX)

S2 Table Interpretation of CCA dimensions in relation to ‘environmental’ gradients (including *P*. *purpuratus* isotopic values) as constraining variables. (DOCX)

S3 Table Macroalgal species scores against CCA dimensions one (~ upwelling influence) and two. Species are listed in rank order by dimension one score.
(DOCX)

S1 Data (ZIP)
