# Supplementary figures and images for: Coastal Upwelling Drives Intertidal Assemblage Structure and Trophic Ecology
Source: PLoS One. 2015 Jul 27;10(7):e0130789. doi: 10.1371/journal.pone.0130789 (PMC4516361; doi:10.1371/journal.pone.0130789)

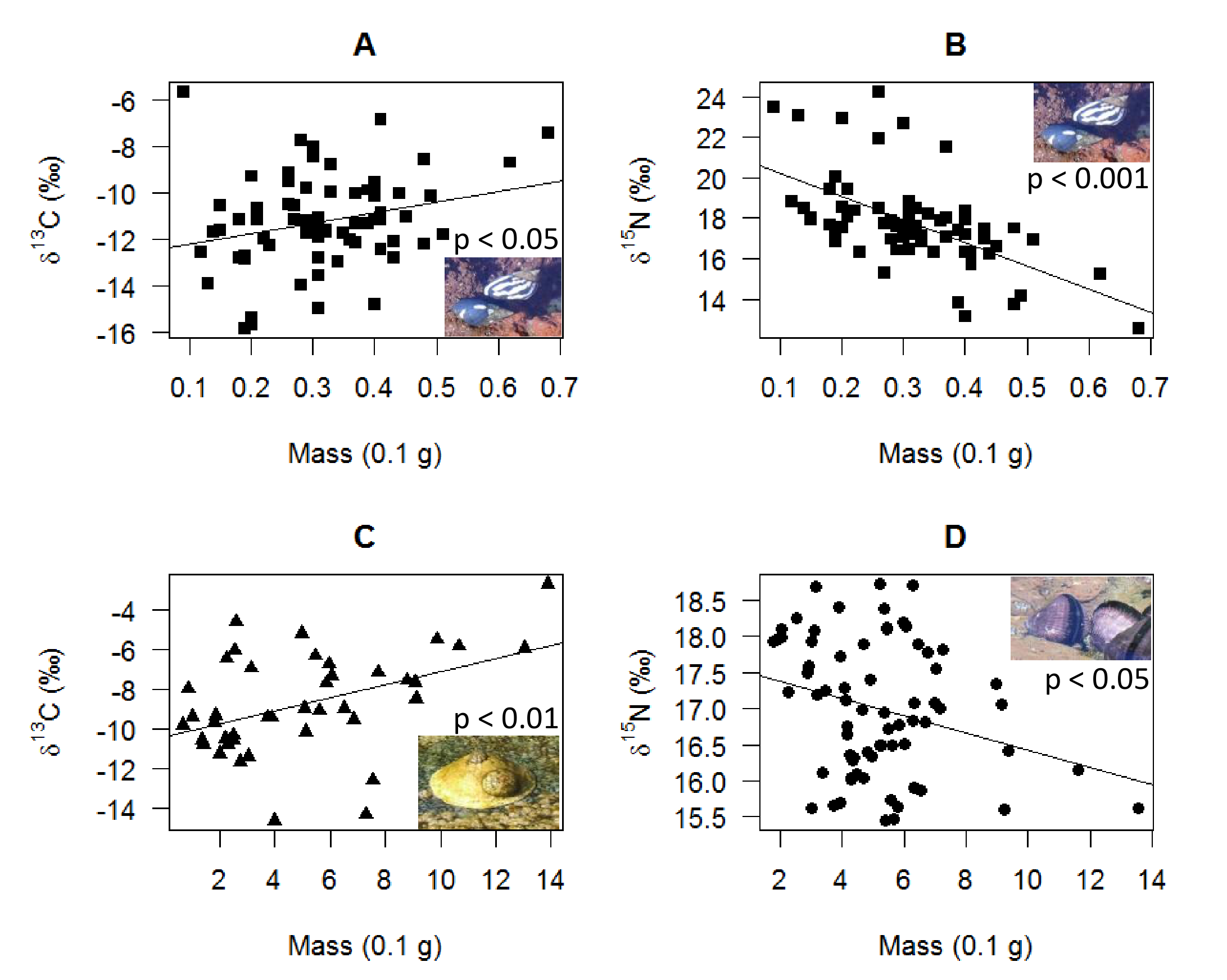

Supplement: S1 Fig — Significant coefficients between individual mass and E. peruviana δ13C and δ15N (A & B, respectively), and S. viridula δ13C (C), and P. purpuratus δ15N (D). Regression residuals were normal. (TIF) [file pone.0130789.s001.tif]
