# Supplementary material for: Coastal Upwelling Drives Intertidal Assemblage Structure and Trophic Ecology
Source: PLoS One. 2015 Jul 27;10(7):e0130789. doi: 10.1371/journal.pone.0130789 (PMC4516361; doi:10.1371/journal.pone.0130789)
Supplement: S1 Table — Significant coefficients between individual mass and E. peruviana δ13C and δ15N (A & B, respectively), and S. viridula δ13C (C), and P. purpuratus δ15N (D). (DOCX) [file pone.0130789.s002.docx]

|  | 1. Response: *E. peruviana* δ^13^C | | | | | |  | |  |
| --- | --- | --- | --- | --- | --- | --- | --- | --- | --- |
|  |  | Coefficients | | Df | F value | R^2^_adj_ | | p |  |
|  | (Intercept) | -12.66 | |  |  |  | | <0.001 |  |
|  | Mass (g) | 4.55 | | 1, 66 | 4.77 | 0.05 | | <0.05 |  |
|  | 1. Response: *E. peruviana* δ^15^N | | | | | |  | |  |
|  |  | | Coefficients | Df | F value | R^2^_adj_ | | p |  |
|  | (Intercept) | | 21.39 |  |  |  | | <0.001 |  |
|  | Mass (g) | | -11.53 | 1, 66 | 35.36 | 0.34 | | <0.001 |  |
|  | 1. Response: *S. viridula* δ^13^C | | | | | |  | |  |
|  |  | | Coefficients | Df | F value | R^2^_adj_ | | p |  |
|  | (Intercept) | | -10.38 |  |  |  | | <0.001 |  |
|  | Mass (g) | | 0.33 | 1, 39 | 8.87 | 0.16 | | <0.01 |  |
|  | 1. Response: *P. purpuratus* δ^15^N | | | | | |  | |  |
|  |  | | Coefficients | Df | F value | R^2^_adj_ | | p |  |
|  | (Intercept) | | 17.62 |  |  |  | | <0.001 |  |
|  | Mass (g) | | -0.12 | 1, 68 | 5.66 | 0.06 | | <0.05 |  |
|  |  | |  |  |  |  | |  |  |

Regression residuals were normal.
