# Supplementary material for: Coastal Upwelling Drives Intertidal Assemblage Structure and Trophic Ecology
Source: PLoS One. 2015 Jul 27;10(7):e0130789. doi: 10.1371/journal.pone.0130789 (PMC4516361; doi:10.1371/journal.pone.0130789)
Supplement: S2 Table — (DOCX) [file pone.0130789.s003.docx]

|  |  | CCA1 | CCA2 | CCA3 | CCA4 | CCA5 | Residual |  |
| --- | --- | --- | --- | --- | --- | --- | --- | --- |
|  | *P. purpuratus* δ^13^C | 0.01 | -0.89 | -0.14 | -0.33 | -0.29 |  |  |
|  | *P. purpuratus* δ^15^N | 0.94 | -0.08 | -0.13 | -0.21 | -0.22 |  |  |
|  | Substratum stability | 0.41 | 0.30 | 0.53 | -0.21 | 0.64 |  |  |
|  | Human impact | -0.72 | -0.12 | -0.54 | -0.20 | 0.37 |  |  |
|  | BayOpen | 0.69 | -0.62 | -0.15 | 0.33 | 0.02 |  |  |
|  |  |  |  |  |  |  |  |  |
|  | EIGENVALUE | 0.20 | 0.19 | 0.09 | 0.08 | 0.03 |  |  |
|  | Cum. Proportion explained | 0.33 | 0.65 | 0.81 | 0.94 | 1.00 |  |  |
|  | df | 1 | 1 | 1 | 1 | 1 | 12 |  |
|  | Chi sq | 0.2 | 0.19 | 0.09 | 0.08 | 0.03 | 0.85 |  |
|  | F value | 2.8 | 2.68 | 1.3 | 1.14 | 0.47 |  |  |
|  | P value | 0.0021 | 0.0009 | 0.1917 | 0.3114 | 0.9707 |  |  |

The top five rows show the biplot scores (~correlations) for ‘environmental’ gradients against the CCA dimensions, followed by the eigenvalues and cumulative proportion explained, and significance as tested by a permutational test (n. perms = 9999), of the CCA dimensions.
