# Supplementary material for: Coastal Upwelling Drives Intertidal Assemblage Structure and Trophic Ecology
Source: PLoS One. 2015 Jul 27;10(7):e0130789. doi: 10.1371/journal.pone.0130789 (PMC4516361; doi:10.1371/journal.pone.0130789)
Supplement: S3 Table — Species are listed in rank order by dimension one score. (DOCX) [file pone.0130789.s004.docx]

|  | Species name | Taxonomic Authority | Dim. 1 Score | Dim. 2 Score | Information content* |  |
| --- | --- | --- | --- | --- | --- | --- |
|  | *Ahnfeltiopsis furcellata* | (C.Agardh) P.C.Silva & DeCew, 1992 | 1.26 | -0.88 | 0.56 |  |
|  | *Nothogenia fastigiata* | (Bory de Saint-Vincent) P.G.Parkinson, 1983 | 1.13 | 0.83 | 0.40 |  |
|  | *Schottera nicaeensis* | (J.V. Lamouroux ex Duby) Guiry & Hollenberg, 1975 | 1.06 | 0.64 | 0.56 |  |
|  | *Stenogramma interruptum* | (C.Agardh) Montagne, 1846 | 0.91 | 0.58 | 0.69 |  |
|  | *Chondrus canaliculatus* | (C.Agardh) Greville, 1830 | 0.86 | -0.38 | 0.80 |  |
|  | *Cladophora herpestica* | (Montagne) Kützing, 1849 | 0.78 | -0.76 | 0.80 |  |
|  | *Gastroclonium cylindricum* | Santelices, I.A.Abbott & Ramírez, 1989 | 0.78 | 2.03 | 0.40 |  |
|  | *Pterosiphonia dendroidea* | (Montagne) Falkenberg, 1901 | 0.68 | -1.00 | 0.40 |  |
|  | *Pyropia columbina* | (Montagne) W.A.Nelson, 2011 | 0.62 | -0.61 | 0.69 |  |
|  | *Lessonia nigrescens* | Bory de Saint-Vincent, 1826 | 0.53 | -0.46 | 0.99 |  |
|  | *Gelidium lingulatum* | Kützing, 1868 | 0.43 | 0.20 | 0.89 |  |
|  | *Ulva linza* | Linnaeus, 1753 | 0.41 | -0.34 | 0.40 |  |
|  | *Ahnfeltiopsis durvillei* | (Bory de Saint-Vincent) P.C.Silva & DeCew, 1992 | 0.39 | 0.74 | 0.69 |  |
|  | *Polysiphonia scopulorum* | Harvey, 1855 | 0.26 | 1.15 | 0.56 |  |
|  | *Ulva compressa* | Linnaeus, 1753 | 0.21 | 1.06 | 0.56 |  |
|  | *Ceramium virgatum* | Roth, 1797 | 0.19 | 0.18 | 0.95 |  |
|  | *Cladophora glomerata* | (Linnaeus) Kützing, 1843 | 0.15 | -0.16 | 0.99 |  |
|  | *Colpomenia tuberculata* | De A.Saunders, 1898 | 0.05 | -0.29 | 0.95 |  |
|  | *Centroceras clavulatum* | (C.Agardh) Montagne, 1846 | 0.03 | 0.77 | 0.95 |  |
|  | *Corallina officinalis* | Linnaeus, 1758 | 0.02 | -0.10 | 0.69 |  |
|  | *Gelidium chilense* | (Montagne) Santelices & Montalva, 1983 | 0.01 | 0.01 | 0.00 |  |
|  | *Hildenbrandia* | Nardo, 1834 | 0.01 | 0.01 | 0.00 |  |
|  | *Ralfsia confusa* | Hollenberg, 1969 | 0.01 | 0.01 | 0.00 |  |
|  | *Ulva rigida* | C.Agardh, 1823 | -0.08 | 0.09 | 0.56 |  |
|  | *Ulva lactuca* | Linnaeus, 1753 | -0.09 | 0.38 | 0.99 |  |
|  | *Ulva prolifera* | O.F.Müller, 1778 | -0.14 | -0.63 | 0.80 |  |
|  | *Colpomenia sinuosa* | (Mertens ex Roth) Derbès & Solier, 1851 | -0.15 | -0.20 | 0.89 |  |
|  | Pinkrock lower |  | -0.18 | -0.38 | 0.69 |  |
|  | *Polysiphonia paniculata* | Montagne, 1842 | -0.33 | -0.55 | 0.80 |  |
|  | *Chaetomorpha linum* | (O.F.Müller) Kützing, 1845 | -0.35 | 0.64 | 0.99 |  |
|  | *Ectocarpus* | Lyngbye, 1819 | -0.39 | -0.10 | 1.00 |  |
|  | *Bryopsis peruviana* | W.R.Taylor, 1947 | -0.52 | 0.43 | 0.69 |  |
|  | *Dictyota dichotoma* | (Hudson) J.V.Lamouroux, 1809 | -0.59 | -0.06 | 1.00 |  |
|  | *Dictyota kunthii* | (C.Agardh) Greville, 1830 | -0.61 | 0.29 | 0.95 |  |
|  | *Petalonia fascia* | (O.F.Müller) Kuntze, 1898 | -0.62 | -0.55 | 0.69 |  |
|  | *Cryptopleura imbricata* | E.Y.Dawson, 1962 | -0.68 | -0.39 | 0.80 |  |
|  | Chondria acrorhizophora | Setchell & N.L.Gardner, 1924 | -0.98 | 0.07 | 0.40 |  |
|  | Red filamentious |  | -1.08 | 0.12 | 0.56 |  |
|  | *Hypnea cenomyce* | J.Agardh, 1851 | -1.10 | 0.04 | 0.80 |  |

Also shown are species’ occurrences as the number of sites each occurred at (complete coverage is 18/18 sites). Rare species (those occurring at only one site) were removed prior to CCA as recommended in Borcard *et al.* [58]. * ‘Information content’ by binomial variance, as *p_i_ (1 – p_i_)*, where *p* is the proportion of the study area occupied by species *i*, as in Lennon et al. [84]; variance is scaled so that a value of 1 shows the maximum capacity to inform a correlation, zero shows zero capacity.
